# The availability of essential medicines for mental healthcare in Sofala, Mozambique

**DOI:** 10.3402/gha.v8.27942

**Published:** 2015-06-15

**Authors:** Bradley H. Wagenaar, Andy Stergachis, Deepa Rao, Roxanne Hoek, Vasco Cumbe, Manuel Napúa, Kenneth Sherr

**Affiliations:** 1 Department of Epidemiology, University of Washington School of Public Health, Seattle, WA, USA; 2 Health Alliance International, Seattle, WA, USA; 3 Department of Global Health, University of Washington School of Public Health, Seattle, WA, USA; 4 Global Medicines Program, University of Washington, Seattle, WA, USA; 5 Department of Psychiatry and Behavioral Sciences, University of Washington, Seattle, WA, USA; 6 Health Alliance International, Beira, Mozambique; 7 Beira Operations Research Center, Ministry of Health, Beira, Mozambique; 8 Department of Mental Health, Ministry of Health, Beira, Mozambique; 9 Psychiatric Services, Department of Medicine, Beira Central Hospital, Beira, Mozambique

**Keywords:** mental health systems, delivery of mental healthcare, pharmaceutical services, Mozambique, health planning

## Abstract

**Objective:**

We assessed the availability of essential medicines for mental healthcare (MH) across levels of the public healthcare system to aid in future systems planning.

**Design:**

Non-expired MH medications were assessed in 24 public health facilities and 13 district warehouses across Sofala Province, Mozambique, from July to August 2014. Medication categories included: antipsychotics, antidepressants, benzodiazepines, antiepileptics and mood stabilizers, and anticholinergics and antihistamines.

**Results:**

Only 7 of 12 (58.3%) district warehouses, 11 of 24 (45.8%) of all health facilities, and 10 of 12 (83.3%) of facilities with trained MH staff had availability of at least one medication of each category. Thioridazine was the most commonly available antipsychotic across all facilities (9 of 24, 37.5%), while chlorpromazine and thioridazine were most common at facilities providing MH care (8 of 12, 66.7%). The atypical antipsychotic risperidone was not available at any facility or district warehouse. Amitriptyline was the most commonly available antidepressant (10 of 12 districts; 12 of 24 overall facilities; 9 or 12 MH facilities). Despite being on the national essential drug list, fluoxetine was only available at one quaternary-level facility and no district warehouses.

**Conclusions:**

Essential psychotropic medicines are routinely unavailable at public health facilities. Current essential drug lists include six typical but no atypical antipsychotics, which is concerning given the side-effect profiles of typical antipsychotics. Ensuring consistent availability of at least one selective serotonin reuptake inhibitor should also be a priority, as they are essential for the treatment of individuals with underlying cardiovascular disease and/or suicidal ideation. Similar to successful task-sharing approaches used for HIV/AIDS, mid-level providers could be retrained and certified to prescribe and monitor first-line psychotropic regimens.

In 2001, The World Health Organization (WHO) published the World Health Report on global mental health, entitled ‘Mental Health – New Understanding, New Hope’ (1). This report outlined 10 recommendations to WHO member countries, with the first two being: provide mental health treatment in primary care and improve the availability of psychotropic drugs globally. In addition, the last recommendation of the report was to support more global mental health research. Now 14 years on from this landmark publication and there remain no peer-reviewed implementation research articles on the distribution, availability, and/or the frequency of stock-outs regarding essential psychotropic medications in public health facilities in Mozambique – a country with a population of 25.8 million (2). More generally, there are few peer-reviewed articles focusing on access, cost, quality, and use of psychotropics in sub-Saharan Africa. This lack of up-to-date data on the availability of essential medicines for mental healthcare inhibits quality patient care, and the development, targeting, and testing of implementation, operations, and quality-improvement approaches to improve care and treatment for mental disorders.

According to the 2010 Global Burden of Disease estimates for Mozambique, mental disorders account for 23.1% of all years lived with disability when considering those aged 15–49 (3). With this large burden of mental illness, Mozambique has an estimated shortage of 236 psychiatrists and 2,389 mental health technicians to achieve basic WHO targets for the provision of mental healthcare (4). Task-sharing approaches using non-specialist healthcare workers have been advocated to overcome the large mental health treatment gap that exists in most low-and middle-income countries (LMICs) (5–8). However, a recent review among a sample of LMICs found that improved access to essential psychotropic medications was considered a basic prerequisite for the success of mental health task-sharing approaches (9).

Mozambique does not have an official approved mental health policy separate from the general health policy or dedicated mental health legislation, and apportions an estimated 0.16% of the national health budget to mental health (10). There are no family- or community-based support organizations for mental disorders in Mozambique (11). Mozambique does, however, have an essential drug list that includes antipsychotics, antidepressants, benzodiazepines, antiepileptics and mood stabilizers, as well as anticholinergic medications (12).

Previous cross-national studies of LMICs (excluding Mozambique) have indicated that nations that have a national mental health plan, family-based organizations participating in drafting mental health legislation, and a higher proportion of the health budget dedicated to mental health are more likely to have consistent availability of essential psychotropic medications (13). In addition, the 2009 WHO Assessment Instrument for Mental Health System (WHO-AIMS) study of mental health systems in 42 LMICs found that, across a sample of African countries, only 14% had at least one psychotropic medicine in each of five categories (antipsychotics, anxiolytics, antidepressants, mood stabilizers, and antiepileptic drugs) available in all public health facilities. Medication cost was also an issue – the median cost in low-income countries of antipsychotic medication was 9% of the minimum wage (13). These data are limited by the fact that WHO-AIMS did not verify the availability of non-expired psychotropic medications at the point of care in the sampled countries, an aim of the present study. In Nigeria, even after a 15-year program focused on the scale-up of mental healthcare treatment in primary care settings, the majority of public health facilities did not have routine availability of essential psychotropic medications (14).

Treatment of epilepsy with first-line antiepileptic drugs, (such as carbamazepine), depression with generic antidepressants (such as amitriptyline or fluoxetine) plus brief psychotherapy, and psychosis with first-generation antipsychotics (such as haloperidol or fluphenazine) have been rated as among the most cost-effective interventions for non-communicable diseases in LMICs (15). Since mental disorders are often chronic in nature, stock-outs or lack of access to medicines in public health facilities can have a large impact on adherence and treatment effectiveness (16). In addition, since many psychotropic medications are subject to illicit use and can be habit-forming, accurate understanding and regulation of the distribution of these drugs in LMICs is essential (17, 18).

Previous work in this same setting of Mozambique that assessed the availability of 15 essential medicines for primary care provision – such as erythromycin, injectable quinine, condoms, and diphtheria, pertussis, and tetanus vaccine – found that stock-outs were common (19). Across three years of cross-sectional facility surveys, over 70% of visits had at least one essential medicine currently unavailable, which often occurred when drug stock was available at the district level (19). These findings suggest that difficulties in transport and communication between districts and health facilities may be the most common reasons for drug stock-outs in this setting.

In 2011, a study using the WHO-AIMS framework in Mozambique found that over 90% of facilities providing outpatient psychiatric care had stock-outs of essential psychotropics in the last year (11). Studies from other LMIC settings, such as India and neighboring South Africa suggest that frequent stock-outs of medicines and/or a lack of availability of medicines for mental healthcare may contribute to attitudes that medications for mental health conditions are ineffective, decrease biomedical care-seeking for mental health conditions, and could increase stigma regarding those suffering from mental disorders (20–22).

The present study represents, to our knowledge, the first examination of verified availability of essential drugs for mental healthcare provision in Mozambique. We aim for this information to help inform approaches to improve prevention, care, and treatment for mental disorders in Mozambique and other settings facing a high burden of mental disorders, and limited resources in the health system.

## Methods

### Study setting and sampling frame

As part of the evaluation framework for a comprehensive health-systems-strengthening intervention, we currently conduct annual service provision assessments (SPAs) in 27 of 156 total health facilities and all 13 district warehouses across Sofala Province, Mozambique (23). These cross-sectional SPAs have been ongoing since 2011, focusing on the availability of non-expired essential medicines and supplies, as well as functional essential equipment for primary healthcare provision. The survey instrument was adapted from the SPA data collection forms used for the demographic and health surveys (24), and included a list of tracer medicines (15 total), supplies (7 total), and functioning equipment (9 total) standardized across the five African Health Initiative countries (25).

In 2011, a two-stage sampling approach was used to select facilities for continued repeated cross-sectional SPA surveys. This sample yielded a total of 26 health facilities (two per district across the province), capturing the largest facility in each district and a randomly selected smaller health center. Only public, Ministry-run facilities were considered for inclusion in the sample. Unfortunately, due to civil unrest, we were unable to travel to three health facilities (two in Machanga district and one in Gorongosa district), and thus they are excluded from the 2014 sample. The quaternary-level Beira Central Hospital was included in our analyses because it is one of the primary providers of mental health services in the province.

Detailed information on the design, sampling frame, study setting of Sofala Province, and the Mozambican supply-chain has been previously published (19). Briefly, medications are delivered to public health facilities either quarterly via a push (kit) mechanism or monthly using a pull (requisition) system. All essential mental health medications in the present study are delivered via the pull system, save diazepam and chlorpheniramine. Over 90% of health services in Mozambique are delivered through public-sector primary care clinics; there is no well-established private-sector healthcare delivery system (26). In addition, few to no private pharmacies exist outside larger provincial capital cities.

In the 2014 iteration of the annual cross-sectional survey, essential medicines for mental healthcare were added, along with questions on the availability of mental health treatment manuals, mental healthcare staffing, and referral networks for mental health. Of the 24 health facilities sampled, 12 provide specialist mental healthcare services, most often staffed by a single psychiatric technician. The most recent essential drug list for Mozambique (published in 2010) includes 397 drug formulations that should be available at all times across Ministry of Health primary care facilities nationally (12).

### Variables, measures, and analyses

We used the WHO model list of essential medicines (27), the Mozambican national pharmaceutical formulary (28), and essential drug list (12), along with discussions with local mental health professionals to develop a list of essential medications for mental healthcare provision that respected both national and international guidelines, as well as local realities in Sofala, Mozambique. General categories of medications included: antipsychotics; antidepressants; benzodiazepines; antiepileptics and mood stabilizers; and anticholinergics. Availability of a specific medication refers to the current and non-expired availability of any formulation of medication delivery, whether tablet, injection, or other.

Availability of non-expired drugs was stratified by district-level availability, overall facility availability, availability at facilities with at least one trained specialist mental healthcare worker (psychiatrist, clinical psychologist, psychiatric technician), and facility type. In addition to absolute availability, we also present data on facility-level availability when drugs are concurrently available at the district-level drug warehouse.

## Results

### Facility descriptive statistics and referral networks

**Fig. 1:**
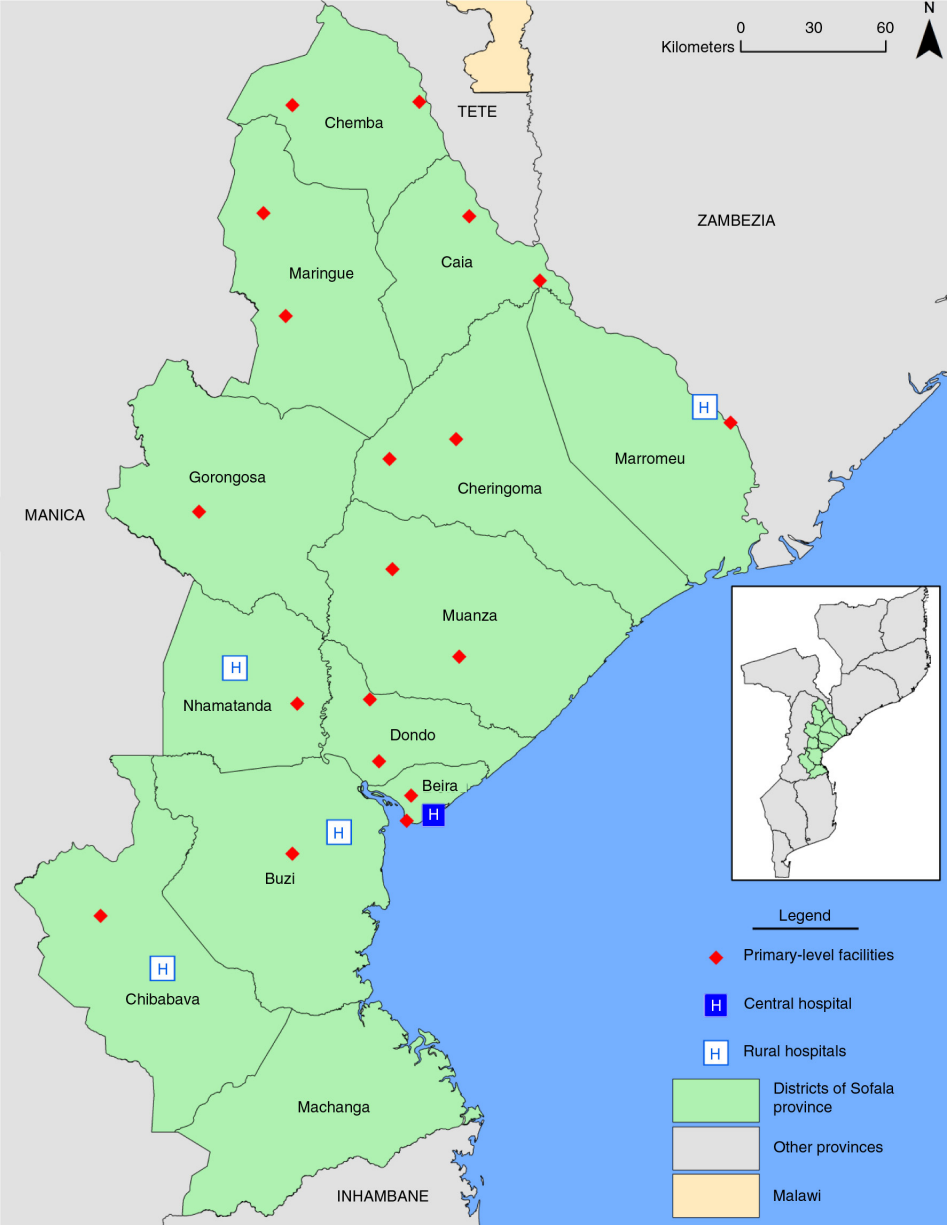
Map of health facilities assessed for availability of essential medicines for mental healthcare and referral networks, Sofala Province, Mozambique, July to August 2014.

Of the 24 facilities, 83% (*n*=20) were rural and the mean number of general outpatient consultations per facility in 2013 was 54,728 (see Table 1; Fig. 1). The majority of facilities in the sample were smaller rural type 2 facilities (*n*=11), followed by larger rural type 1 facilities (*n*=6) and rural hospitals (*n*=4). Only one facility (4.2%) did not have a health professional who could diagnose and prescribe medications for mental healthcare conditions. The vast majority of facilities had not referred a patient for mental health issues (*n*=21 facilities, 87.5%) or suicidal thoughts (*n*=20 facilities, 83.3%) in the last 30 days (Table 1).

**Table 1:** Characteristics of 24 health facilities surveyed for availability of mental health medications, Sofala Province, Mozambique, July to August, 2014

Characteristic	All facilities, *n* (%) unless noted	Facilities providing specialized mental health services, *n* (%) unless noted
Total facilities	24 (100)	12 (50.0)^a^
Rural facility location	20 (83.3)	8 (66.7)
No. of general outpatient consultations in 2013 (mean, SD)	54,728 (46,904)	87,519 (45,240)
Availability of mental health treatment manual	9 (37.5)	8 (66.7)
Type of health facility		
Central Hospital	1 (4.2)	1 (8.3)
Urban Health Center – Type A	2 (8.3)	2 (16.7)
Rural Hospital	4 (16.7)	4 (33.3)
Rural Health Center – Type 2	11 (45.8)	0 (0)
Rural Health Center – Type 1	6 (25.0)	5 (41.7)
Number of patients referred for mental health issues in last 30 days (mean, SD)	0.17 (0.48)	0.33 (0.65)
0	21 (87.5)	9 (75.0)
1	2 (8.3)	2 (16.7)
2	1 (4.2)	1 (8.3)
Number of patients referred for suicidal thoughts in last 30 days (mean, SD)	0.17 (0.57)	0.33 (0.65)
0	20 (83.3)	11 (91.7)
1	2 (8.3)	0 (0)
2	2 (8.3)	1 (8.3)
Number of professionals who can prescribe medications for mental health (mean, SD)	1.7 (0.98)	3.1 (2.5)
0	1 (4.2)	0 (0)
1	8 (33.3)	4 (33.3)
2	9 (37.5)	3 (25.0)
3 +	6 (25.0)	5 (41.7)

a Percentages out of total number of facilities (24).

### District warehouse availability of essential medicines for mental healthcare

Of 12 district warehouses, only 58.3% (*n*=7) had current availability of at least one medication of each category for mental healthcare provision (see Table 2). Carbamazepine was the medication most available, with 91.7% (*n*=11) of warehouses having current availability, followed by amitriptyline (83.3% availability, *n*=10) and diazepam (75.0% availability, *n*=9). No district warehouses had current availability of risperidone, decanoate of fluphenazine, maprotiline, fluoxetine, midazolam, chlordiazepoxide, lithium carbonate, or phenytoin. Haloperidol, chlorpromazine, and thioridazine were all available at 41.7% of warehouses (*n*=5). The most commonly available anticholinergics or antihistamines were promethazine and diphenhydramine, both available at 66.7% of warehouses (*n*=8; Table 2).

**Table 2:** Availability of non-expired mental health medications at district-level warehouses and health facilities, stratified by level of health facility, and whether a given facility has specialist mental health staff, Sofala Province, Mozambique, July to August, 2014

Medication category	Specific medication*	On Ministry of Health ‘essential drug’ list	Lowest-level provider allowed to prescribe^a^	District-level warehouse availability *n* (%)	Overall facility availability *n* (%)	Facility with specialist mental health staff *n* (%)	Rural type 2 *n* (%)	Rural type 1 *n* (%)	Urban type A *n* (%)	Rural hospital *n* (%)	Central hospital *n* (%)
	Total			12 (100.0)	24 (100.0)	12 (50.0)^b^	11 (45.8)^b^	6 (25.0)^b^	2 (8.3)^b^	4 (16.7)^b^	1 (4.2)^b^
Antipsychotics	Haloperidol	Y	M.D. or Specialist	5 (41.7)	8 (33.3)	7 (58.3)	0 (0)	4 (66.7)	1 (50.0)	2 (50.0)	1 (100.0)
	Chlorpromazine	Y	M.D. or Specialist	5 (41.7)	8 (33.3)	8 (66.7)	0 (0)	3 (50.0)	1 (50.0)	3 (75.0)	1 (100.0)
	Fluphenazine	Y	M.D. or Specialist	3 (25.0)	5 (20.8)	5 (41.7)	0 (0)	2 (33.3)	1 (50.0)	1 (25.0)	1 (100.0)
	Trifluoperazine	Y	M.D. or Specialist	2 (16.7)	6 (25.0)	6 (50.0)	0 (0)	2 (33.3)	1 (50.0)	2 (50.0)	1 (100.0)
	Thioridazine	Y	M.D. or Specialist	5 (41.7)	9 (37.5)	8 (66.7)	0 (0)	3 (50.0)	2 (100.0)	4 (100.0)	0 (0)
	Risperidone	N	n/a	0 (0)	0 (0)	0 (0)	0 (0)	0 (0)	0 (0)	0 (0)	0 (0)
	Decanoate of fluphenazine	Y	M.D. or Specialist	0 (0)	4 (16.7)	4 (33.3)	0 (0)	0 (0)	1 (50.0)	2 (50.0)	1 (100.0)
Antidepressants	Amitriptyline	Y	M.D. or Specialist	10 (83.3)	12 (50.0)	9 (75.0)	2 (18.2)	5 (83.3)	2 (100.0)	2 (50.0)	1 (100.0)
	Imipramine	Y	M.D. or Specialist	3 (25.0)	7 (29.2)	7 (58.3)	0 (0)	3 (50.0)	1 (50.0)	2 (50.0)	1 (100.0)
	Maprotiline	Y	M.D. or Specialist	0 (0)	1 (4.2)	1 (8.3)	0 (0)	0 (0)	0 (0)	0 (0)	1 (100.0)
	Fluoxetine	Y	M.D. or Specialist	0 (0)	1 (4.2)	1 (8.3)	0 (0)	0 (0)	0 (0)	0 (0)	1 (100.0)
Benzodiazepines	Diazepam	Y	CHW	9 (75.0)	18 (75.0)	11 (91.7)	6 (54.6)	5 (83.3)	2 (100.0)	4 (100.0)	1 (100.0)
	Midazolam	N	n/a	0 (0)	1 (4.2)	1 (8.3)	0 (0)	0 (0)	0 (0)	0 (0)	1 (100.0)
	Chlordiazepoxide	Y	Nurse	0 (0)	0 (0)	1 (8.3)	0 (0)	1 (5.6)	0 (0)	0 (0)	0 (0)
Antiepileptics and	Carbamazepine	Y	M.D. or Specialist	11 (91.7)	14 (58.3)	10 (83.3)	3 (27.3)	5 (83.3)	2 (100.0)	4 (100.0)	0 (0)
mood stabilizers	Lithium carbonate	N	n/a	0 (0)	0 (0)	0 (0)	0 (0)	0 (0)	0 (0)	0 (0)	0 (0)
	Sodium valproate	Y	M.D. or Specialist	8 (66.7)	7 (29.2)	7 (58.3)	0 (0)	2 (33.3)	2 (100.0)	2 (50.0)	1 (100.0)
	Phenytoin	Y	M.D. or Specialist	0 (0)	2 (8.3)	2 (16.7)	0 (0)	0 (0)	0 (0)	1 (25.0)	1 (100.0)
	Phenobarbital	Y	Med. Technician	6 (50.0)	15 (62.5)	9 (75.0)	5 (45.5)	4 (66.7)	2 (100.0)	4 (100.0)	0 (0)
Anticholinergics and	Promethazine	Y	M.D. or Specialist	8 (66.7)	11 (45.8)	11 (91.7)	0 (0.0)	5 (83.3)	1 (50.0)	4 (100.0)	1 (100.0)
Antihistamines	Biperiden	Y	M.D. or Specialist	2 (16.7)	6 (25.0)	6 (50.0)	0 (0.0)	1 (16.7)	2 (100.0)	2 (50.0)	1 (100.0)
	Chlorpheniramine	Y	CHW	6 (50.0)	19 (79.2)	10 (83.3)	8 (72.7)	6 (100.0)	1 (50.0)	3 (75.0)	1 (100.0)
	Diphenhydramine	Y	CHW	8 (66.7)	17 (70.8)	11 (91.7)	5 (45.5)	6 (100.0)	2 (100.0)	4 (100.0)	0 (0)
At least one of each category			7 (58.3)	11 (45.8)	10 (83.3)	0 (0)	5 (83.3)	2 (100.0)	3 (75.0)	1 (100.0)	

* Availability of medication refers to the current and non-expired availability of any formulation of medication delivery, whether tablet, injection, or other.

a M.D. or Specialist = Medical Doctor, psychiatrist, or psychiatric technician; CHW = Community Health Worker

b Percentages are out of total number of facilities (24) for all percentages excluding the column for district-level warehouse availability, where there were 12 district warehouses surveyed. For clarification purposes, there were 24 total facilities and 12 district warehouses included in the sample. Twelve of the overall 24 facilities had trained specialist mental health staff present (either a psychiatric technician or psychiatrist). Eleven of the 24 were rural type 2, 6 of the 24 were rural type 1, and so on across the other facility classifications. Rural type 2 facilities serve catchment areas of 7,500–20,000 population, have an average of 4 healthcare staff, and conduct outpatient, prenatal, well-child, and maternity services. Rural type 1 facilities serve catchment areas of 16,000–35,000, have an average of 13–16 healthcare staff, and conduct outpatient, prenatal, well-child, and maternity services. Urban type A facilities serve catchment areas of 40,000–100,000, have 26–36 health staff, and conduct all main primary health care services, including x-ray capabilities, oral healthcare, emergency care including first-aid and minor surgery, and inpatient beds. Rural hospitals serve catchment areas of 150,000–900,000, have 60–100 healthcare staff, and provide specialized care including an advanced laboratory, radiology capabilities, blood banks, and major surgical wards. Central hospitals serve catchment areas of over 2 million, have a large staff of healthcare workers including specialists, and provide quaternary-level healthcare with specialized care such as neurology, cardiology, neuro-surgery, oncology, psychiatry, etc.

### Availability of essential medicines for mental healthcare across all facilities

Of 24 health facilities, only 45.8% (*n*=11) had current availability of at least one medication of each category for mental healthcare provision (see Table 2). All antipsychotic medications had less than 38% availability, with thioridazine being most available (37.5%, *n*=9), followed by haloperidol and chlorpromazine (both 33.3%, *n*=8). Amitriptyline was available in half of the facilities (*n*=12) and diazepam was available in 75.0% (*n*=18) of facilities. Phenobarbital was the most available antiepileptic drug (available in 62.5% of facilities, *n*=15), followed by carbamazepine (58.3%, *n*=14). Chlorpheniramine was available in 79.2% of facilities (*n*=19), followed by diphenhydramine (70.8% of facilities, *n*=17).

**Table 3:** Stock-outs of essential mental health medicines when available at district distribution point, Sofala Province, Mozambique, July to August, 2014

Medication category	Specific medication	No stock at facilities providing specialized mental healthcare when available at district level, *n* (%)
	Total	12 (50)^a^
Antipsychotics	Haloperidol	1 (8.3)
	Chlorpromazine	2 (16.7)
	Fluphenazine	1 (8.3)
	Trifluoperazine	0 (0)
	Thioridazine	1 (8.3)
	Risperidone	0 (0)
	Decanoate of fluphenazine	0 (0)
Antidepressants	Amitriptyline	2 (16.7)
	Imipramine	2 (16.7)
	Maprotiline	0 (0)
	Fluoxetine	0 (0)
Benzodiazepines	Diazepam	0 (0)
	Midazolam	0 (0)
	Chlordiazepoxide	0 (0)
Antiepileptics and mood stabilizers	Carbamazepine	2 (16.7)
	Lithium carbonate	0 (0)
	Sodium valproate	4 (33.3)
	Phenytoin	0 (0)
	Phenobarbital	0 (0)
Anticholinergics	Promethazine	1 (8.3)
	Biperiden	1 (8.3)
	Chlorpheniramine	0 (0)
	Diphenhydramine	0 (0)

a Percentage out of total number of facilities (24).

### Facility availability of essential medicines for mental healthcare at facilities with specialized mental healthcare services

Over 83% (*n*=10) of the 12 facilities with specialized mental healthcare services had current availability of at least one medication from each category for mental healthcare provision (see Table 2). No medication was available at all facilities. The most commonly available medications were diazepam, carbamazepine, promethazine, and diphenhydramine, which were all available at 11 of 12 facilities (91.7%). No facilities had availability of risperidone or lithium carbonate and only one facility had availability of fluoxetine. Chlorpromazine and thioridazine were the most commonly available antipsychotics (66.7% of facilities, *n*=8), and amitriptyline was the most commonly available antidepressant (75% of facilities, *n*=9).

### Facility availability of essential medicines stratified by facility type

No rural type 2 facilities had current availability of any antipsychotic medications (Table 2). At higher-level facilities, all classifications had greater than 75% availability of at least one drug from each category for care provision.

### Facility availability of mental health medications when available at district distribution point

For facilities providing specialized mental health services, there were few instances of medicines unavailable at the facility level with concurrent availability at the district warehouse. Sodium valproate was unavailable at 33.3% (*n*=4) of the 12 facilities while available at the district distribution point (Table 3).

## Discussion

In this study of public-sector health facilities and a census of district-level drug warehouses, we found that essential medicines for the provision of mental healthcare were routinely unavailable. Medications were more often available at facilities with mental health specialists offering specialized outpatient or other types of mental healthcare, although 96% of all health facilities surveyed had at least one provider who could prescribe medications for mental health conditions. In contrast to previous assessments (19) of the availability of other classes of drugs (antibiotics, vaccines, antimalarials) for primary healthcare provision, where the majority of facility stock-outs occurred while drugs were available at district warehouses, the majority of psychotropic drug stock-outs appear to be due to more distal upstream factors – such as lack of drug stock at provincial or national levels – instead of mismanagement or delays in distribution of existing drugs from district warehouses to health facilities.

The Mozambican essential medicine list currently includes six typical antipsychotics, but no atypical antipsychotics. Not surprisingly, no district warehouses or health facilities had availability of risperidone, a WHO-recommended essential atypical antipsychotic. With regard to antidepressants, although the national essential drug list includes two tricyclics, one tetracyclic, and one selective serotonin reuptake inhibitor (SSRI), only the quaternary-level Central Hospital had availability of any antidepressants other than tricyclics. These findings give pause for a number of reasons. First, no single typical antipsychotic was available at greater than 67% of facilities offering specialized psychiatric services (no more than 37.5% of all facilities) or more than 42% of district warehouses. While it is laudable to strive for a diversity of antipsychotics to allow tailoring of medication regimens or shifting of treatments if side-effects develop, the inconsistent availability of any regimen is concerning. In reality, the current prioritization of six typicals, all haphazardly available, likely forces providers to shift regimens frequently and often due to lack of stock, even if a given formulation has proven effective for a given patient.

Going forward, we recommend prioritizing the consistent availability of haloperidol, fluphenazine, and chlorpromazine, and the addition of the atypical antipsychotic risperidone. Risperidone was added to the WHO essential medicine list in 2013, cited as more effective in the treatment of psychosis, schizophrenia, and bipolar compared to typical antipsychotics (29). In addition, it has fewer extrapyramidal side-effects compared to typical antipsychotics, and thus represents an ‘essential’ alternative for those who cannot tolerate older medications. This may be particularly important as very high rates of anticholinergic medication use to counter side-effects of typical antipsychotics have been cited in Mozambique (30). Available literature on the cost-effectiveness of risperidone indicates that it is either cost-neutral or perhaps cost-saving compared to typical antipsychotics, such as haloperidol, for the treatment of schizophrenia, schizoaffective disorder, and psychosis (29), although the vast majority of cost-effectiveness studies have been conducted in high-income settings. More efforts are needed to characterize the cost-effectiveness of atypical antipsychotics, including risperidone, in LMICs.

The lack of availability of SSRI antidepressants is also concerning as they are the first choice for the treatment of depression for individuals with ideas, plans, or acts of self-harm (31); this may be especially important given Mozambique was recently estimated to have the seventh highest suicide rate in the world, and the highest in Africa (32). Fluoxetine, in particular, is necessary for the treatment of adolescent and older adults with depressive illness, along with individuals with underlying cardiovascular disease or abnormalities, since tricyclics have consistently been associated with increased cardiovascular risk (31). Similar to antipsychotic medication in Mozambique, we recommend a more focused approach to ensure reliable access to both amitriptyline (a tricyclic) and fluoxetine (a SSRI) across all public facilities.

No antidepressants or antipsychotics were routinely available at smaller rural health facilities, referred to as rural type 2 or smaller facilities, and phenobarbital for the treatment of epilepsy was available at less than half of these facilities. Of approximately 1,300 public health facilities nationwide, around 1,000 (or 75%) are smaller rural health centers serving catchment areas of 7,500–20,000 people and providing routine outpatient, prenatal, maternity, and well-child services. These facilities are the bedrock of primary care provision in Mozambique, especially for rural populations far from district capitals and major transport corridors, who do not have access to private pharmacies if medicines are unavailable at public-sector clinics. After effective care is ensured at larger referral facilities, a next step in moving beyond the current situation where only 0.3% of the population has reliable access to mental health services is to extend access to include these smaller, more rural health facilities (33). Given that not a single rural type 2 facility referred a single mental health patient in the past 30 days, a near-term strategy could be to strengthen mental health and self-harm referral networks. In the longer term, the mental healthcare service should take a lesson from the rapid and effective scale-up of antiretroviral treatment for HIV/AIDS and provide re-training and certification for nurses and other mid-level providers (such as medical technicians in the Mozambican context) to prescribe and monitor first-line regimens for outpatient primary mental healthcare (34, 35). Once complete, first-line antipsychotic and antidepressant medications could be included in the routinely distributed push (kit) system nationally.

Given the current continued mental health treatment gap in Mozambique, the current model whereby medical doctors or specialists (including psychiatric technicians) are the only providers allowed to prescribe any antipsychotic or antidepressant should be reconsidered. Flexibility in prescribing regulations by cadre is especially important given the inconsistent availability of any specific psychotropic medications found in this study. For example, under the present system, medical technicians are allowed to prescribe phenobarbital, although depending on the level or type of health facility, carbamazepine may be the only available anti-epileptic. This could lead to a situation where a medical technician cannot continue treating a patient under official guidelines because phenobarbital is unavailable and they are not allowed to prescribe sodium valproate or carbamazepine. Issues such as these beg answering as essential drug lists and provider regulations are debated in Mozambique and other similar LMICs.

This study is not without limitations. We analyzed medication availability among a non-random sample of facilities in one province of Mozambique, and thus the findings may not be representative of facilities across Sofala Province or other areas nationally. Although the facilities selected here were among the largest in the province and contained the majority of facilities currently providing specialized mental healthcare in the province, availability was also assessed cross-sectionally; therefore, patterns may not represent availability over longer time periods. Furthermore, availability was defined as having current and non-expired drug of any formulation, whether injectable or tablet, and therefore the patterns outlined here likely over-represent the real availability of a given specific pharmaceutical formulation. Lastly, while availability is an essential precursor to effective treatment, we did not assess quality or dosage information which should be considered for future study.

These findings also have a number of strengths. Medication availability was physically assessed by visiting each facility and verifying the presence of non-expired medications – not through self-report or an external questionnaire. Second, availability was assessed at a census of all district warehouses, along with a sample including all levels of public facilities, from small rural facilities to the quaternary-level central hospital. Finally, a thorough and comprehensive list of mental health medications was assessed, triangulated between the national essential drug list, the national formulary, as well as WHO essential lists.

## Conclusions

In this study of public-sector Ministry of Health facilities and district drug warehouses in Sofala, Mozambique, we found that essential psychotropic medicines were often unavailable. More specifically, no more than half of district warehouses had current availability of any essential typical antipsychotic and no district or individual facilities had availability of the atypical antipsychotic risperidone. Even though listed on national and international essential medicine lists, fluoxetine was not available at any district warehouse, and only available at the Central Hospital. We recommend updating essential medicine lists and supply chains to prioritize consistent availability of the typical antipsychotics of haloperidol, fluphenazine, and chlorpromazine, with the addition of the atypical antipsychotic risperidone. We also recommend a focused approach to ensure reliable access to both a tricyclic – amitriptyline, and an SSRI – fluoxetine. As essential medicine lists are updated, restrictions around who can prescribe psychotropic medications should additionally be reviewed.

## Ethical approval

This study was approved by the institutional review boards at the University of Washington and the Mozambican National Institute of Health.
